# Identifying patient subgroups with different trends of patient-reported outcomes (PROMs) after elective knee arthroplasty

**DOI:** 10.1186/s12891-023-06373-2

**Published:** 2023-06-03

**Authors:** Davide Golinelli, Alberto Grassi, Francesco Sanmarchi, Dario Tedesco, Francesco Esposito, Simona Rosa, Paola Rucci, Marilina Amabile, Monica Cosentino, Barbara Bordini, Maria Pia Fantini, Stefano Zaffagnini

**Affiliations:** 1 Department of Biomedical and Neuromotor Sciences (DIBINEM), Alma Mater Studiorum - University of Bologna, Via San Giacomo 12, Bologna, 40126 Italy; 2 IIa Clinica Ortopedica e Traumatologica, IRCCS Istituto Ortopedico Rizzoli, Via Pupilli 1, Bologna, 40136 Italy; 3 Directorate-General Personal Care, Health and Welfare, Emilia-Romagna Region, Viale Aldo Moro, 21, Bologna, 40127 Italy; 4 Laboratorio di Tecnologia Medica, IRCCS Istituto Ortopedico Rizzoli, Via Pupilli 1, Bologna, 40136 Italy

**Keywords:** Patient-reported outcome measures, PROMs, Quality improvement, Arthroplasty, Knee, Implant registry

## Abstract

**Background:**

Patient-reported outcome measures (PROMs) are increasingly being used to assess the effectiveness of elective total knee arthroplasty (TKA). However, little is known about how PROMs scores change over time in these patients. The aim of this study was to identify the trajectories of quality of life and joint functioning, and their associated demographic and clinical features in patients undergoing elective TKA.

**Methods:**

A prospective, cohort study was conducted, in which PROMs questionnaires (Euro Quality 5 Dimensions 3L, EQ-5D-3L, and Knee injury and Osteoarthritis Outcome Score Patient Satisfaction, KOOS-PS) were administered to patients at a single center undergoing elective TKA before surgery, and at 6 and 12 months after surgery. Latent class growth mixture models were used to analyze the patterns of change in PROMs scores over time. Multinomial logistic regression was used to investigate the association between patient characteristics and PROMs trajectories.

**Results:**

A total of 564 patients were included in the study. The analysis highlighted differential patterns of improvement after TKA. Three distinct PROMs trajectories were identified for each PROMs questionnaire, with one trajectory indicating the most favorable outcome. Female gender appears to be associated with a presentation to surgery with worse perceived quality of life and joint function than males, but also more rapid improvement after surgery. Having an ASA score greater than 3 is instead associated with a worse functional recovery after TKA.

**Conclusion:**

The results suggest three main PROMs trajectories in patients undergoing elective TKA. Most patients reported improved quality of life and joint functioning at 6 months, which then stabilized. However, other subgroups showed more varied trajectories. Further research is needed to confirm these findings and to explore the potential clinical implications of these results.

**Supplementary Information:**

The online version contains supplementary material available at 10.1186/s12891-023-06373-2.

## Background

Total knee arthroplasty (TKA) is a common surgical procedure for individuals with severe knee osteoarthritis [1]. TKA has been shown to be effective in relieving pain and improving physical function in patients, with reported success rates ranging from 70 to 90% [2]. While TKA is often successful in relieving pain and improving mobility, it is important to understand the full impact of the surgery on patients’ lives. Patient-reported outcome measures (PROMs) can provide valuable information on patients’ perspective of their health status, functioning, and quality of life, and are increasingly being used to evaluate the effectiveness of healthcare interventions [3].

In the last few years, PROMs have been used to assess patients’ experiences before and after TKA, including pain levels, physical function, and overall satisfaction with surgery [4], to identify areas for improvement, and to guide clinical decision-making [5, 6].

The Organisation for Economic Cooperation and Development (OECD) Patient-Reported Indicators Surveys (PaRIS) Initiative has been promoted to develop internationally comparable PROMs for use in the evaluation of healthcare interventions for chronic conditions from patients’ perspective [7]. The PaRIS Initiative has focused on the systematic collection of two PROMs indicators (Euro Quality 5 Dimensions 3L, EQ-5D-3L, and Knee injury and Osteoarthritis Outcome Score Patient Satisfaction (KOOS-PS) in patients undergoing elective knee arthroplasty [8–11]. The decision to use these selected PROMs measures was based on consensus among the OECD’s PaRIS Initiative investigators, as reported in a previous publication [12].

The IRCCS Rizzoli Orthopedic Institute (IOR), a third-level single-specialty orthopedic hospital located in the Emilia-Romagna region, and member of the International Society of Orthopaedic Centers (ISOC), was selected as one of the centers to launch the PaRIS Initiative in Italy. The aim of the initiative was to accelerate the adoption and reporting of validated, standardized, and internationally comparable patient-reported indicators. Of note, more than 60% of patients admitted to IOR for knee replacement surgery come from other Italian regions or other countries. Therefore, the study sample can be considered nationally representative.

### Rationale and aim of this study

Despite the overall effectiveness of TKA, several patients do not experience the expected improvements in pain, physical function, and quality of life within the first year post-surgery. PROMs provide valuable information on the patient’s subjective experiences on these outcomes. The aims of this study are to search for subgroups of patients undergoing TKA with distinct trajectories of functioning and quality of life, and to identify potential predictors of these trajectories. Characterizing subgroups of patients with differential outcomes may inform the development of targeted interventions to enhance patient care.

## Methods

### Study design and data collection

The PaRIS-IOR study is a prospective, single-site cohort investigation that began on January 1, 2019.

Patients who underwent elective TKA between January 1st and December 31st, 2019, constituted the baseline population for this study. Data collected included patients’ demographics, the pathology leading to joint replacement, details of the surgical procedures, in-hospital complications, and implant characteristics. Specifically, the following features were collected and analyzed: (i) patient characteristics and profiles, including age and sex distribution, body mass index (BMI), Elixhauser comorbidity index (ECI) [13], American Society of Anesthesiologists (ASA) score, and modified chronic disease score (M-CDS) [14] for clinical severity; (ii) the PROMs questionnaires.

The ECI is a comorbidity index based on International Classification of Diseases (ICD) diagnosis codes and is obtained as an unweighted count of comorbid conditions [13]. The ASA score is a system for evaluating the fitness of patients before surgery, with categories ranging from 1 (healthy person) to 6 (declared brain-dead person whose organs are being removed for donor purposes). The M-CDS [14] is a weighted chronic disease score based on 18 comorbid conditions derived from drug prescriptions and was developed as a prognostic score for 1-year mortality. It is divided into 6 classes (0–1, 2, 3–4, 5–6, 7–9, ≥ 10).

The inclusion criteria for this study were age between 18 and 95 years and elective TKA, while exclusion criteria included severe cognitive impairment, arthroplasty for musculoskeletal cancer, ineligibility for surgical procedures, and TKA in the 12 months prior to enrollment. Detailed information on the study protocol, inclusion and exclusion criteria, and other information can be found in previous publications [12]. This study adheres to the STROBE reporting guidelines for observational studies [15]. Informed consent was obtained from all subjects and/or their legal guardian(s).

The IOR also hosts the Registry of Orthopedic Prosthetic Implants (RIPO), and PROMs data were linked with information routinely collected by the RIPO [16] as well as other regional administrative data (such as hospital discharge records) to track patients’ medical histories and define their health profiles.

### PROMs questionnaires

PROMs questionnaires were administered to patients on the list for elective TKA to assess their quality of life (using the EQ-5D-3 L [17]) and joint-specific functional outcomes (using the KOOS-PS [18]). These questionnaires were administered by specifically trained researchers within 30 days before surgery. Follow-up questionnaires were mailed to patients 6 and 12 months after surgery.

The EQ-5D-3 L, a widespread measure of health-related quality of life, was used to measure five dimensions (mobility, self-care, usual activities, pain/discomfort, and anxiety/depression) on three levels (no problems, some problems, and extreme problems), with reference to the current day. The scores of the five dimensions range from − 0.594 (worst) to 1.0 (best). In addition, the EQ-5D-3 L includes a visual analog scale (VAS) to rate the overall health status from 0 (worst imaginable health) to 100 (best imaginable health) [19]. The validated Italian version of the EQ-5D-3 L was utilized [20].

The KOOS physical function short-form (KOOS-PS), a 7-questions standardized questionnaire, was used to assess the level of function concerning rising from bed, putting on socks, rising from sitting, bending to floor, twisting on the injured knee, kneeling, and squatting in the last week. Each item rates the difficulty experienced on a 5-point scale from ‘none’ to ‘extreme’. The total KOOS-PS score ranges from 0 to 100, with 0 being the worst and 100 being the best functioning for TKA patients. For patients undergoing a total knee replacement, the internal consistency was 0.89, confirming that the KOOS-PS represents a homogeneous construct. Further, construct validity was supported with a correlation of 0.90 with the PF-subscale of the WOMAC. Finally, KOOS-PS is a responsive measure with a standardized response mean (SRM) of 1.4 [21]. The Italian-validated version of the Knee injury and Osteoarthritis Outcome Score physical function short-form was employed [18].

### Statistical analysis

Baseline demographic and clinical characteristics were summarized using mean and standard deviation, median and interquartile range, or absolute and percentage frequencies as appropriate. Patients lost to follow-up were compared with those who completed the study (completers) at 6 and 12 months. The comparison was based on age, gender, BMI, ASA score, and primary diagnosis. This was done to determine whether the completers were representative of the baseline sample. Complete information about variable distributions and missing data can be found in the Supplementary material. Continuous variables were compared between groups using t-tests, and categorical variables were compared using chi-square tests or Fisher’s exact tests as appropriate. Spearman’s correlation coefficient was used to analyze the relationship among PROMs scale scores. Multicollinearity of variables was assessed using the variance inflation factor (VIF). A significance level of 0.05 was adopted.

#### Latent class growth analysis and growth mixture model

Latent class growth analysis (LCGA) was performed as an initial modeling step to identify subgroups of patients with different trajectories of functioning and quality of life from pre-surgery to 12 months following total knee replacement. LCGA is a type of Growth Mixture Modeling (GMM) in which the variance and covariance estimates for the growth factors within each class are assumed to be fixed at zero [22]. This assumption implies that all individual growth trajectories within a class are homogeneous. This technique allows the identification of distinct subgroups that follow a similar pattern of change over time, making it suitable for analyzing longitudinal data [23]. Other longitudinal methodologies, such as conventional growth models, assume that individuals come from a single population and that a single trajectory can adequately summarize the entire population. They also assume that covariates that affect the growth factors influence individuals in the same way. However, there are theoretical reasons to believe that a single growth trajectory would be an oversimplification of the complex growth patterns that may characterize changes among members of different groups, particularly in clinical populations of older adults.

The LCGA method was employed to handle missing data at 6 and 12 months using the full information maximum likelihood algorithm, and to estimate trajectories for the complete set of patients [24, 55]. Standard model fit indices, including the Akaike Information Criteria (AIC) and Bayesian Information Criterion (BIC), were used to identify the best-fitting models. These indices do not have predefined cut-offs and can only be interpreted when comparing different models. A lower AIC and BIC indicate a better fit. Other indices used included entropy (values close to 1.0 denoting excellent fit), at least 1% total count in a class, and high posterior probabilities. In addition, the Vuong-Lo-Mendell-Rubin likelihood ratio test was used to determine the number of classes. The best model was chosen based on trajectories prevalence, goodness of fit indices, overall classification accuracy, and clinical meaningfulness (i.e., substantive interpretation of the trajectories).

Because the LCGA is a very constrained model, assuming that all variances of growth factors are equal (in other words that all individuals in a latent class have the same trajectory), growth mixture models were then employed to estimate separate growth trajectories for each latent class identified by LCGA [25]. Individuals were assigned to the most likely latent class based on posterior probabilities. Finally, the demographic and clinical predictors of the latent classes in GMM using the R3STEP approach were analyzed [26]. This approach accounts for measurement error in the class assignment process and prevents covariates from influencing the definition of class membership. Specifically, the following demographic and clinical variables that are routinely recorded in administrative databases or in the registry were included: age, sex (with male as the reference category), BMI (with normal weight/underweight as the reference category), diagnosis (with osteoarthrosis versus other diagnoses as the reference category), and ASA score (with ASA < 3 as the reference category).

Patients were classified into subgroups based on their trajectories for two PROMs indicators: the KOOS-PS and the EQ-5D-3 L. The KOOS-PS was selected to measure patients’ reported functioning and mobility, while the EQ-5D-3 L was chosen to measure their reported quality of life. We then analyzed differences in demographic and clinical characteristics between the subgroup of patients with worse reported outcomes on both PROMs indicators and the other subgroups using multinomial logistic regression.

All statistical analyses were conducted using SPSS version 28.0 and MPlus version 8.7.

### Protocol registration

Protocol version (1.0) and trial registration data are available on the platform www.clinicaltrial.gov with the identifier NCT03790267, posted on December 31, 2018.

## Results

### Study population

**Fig. 1:**
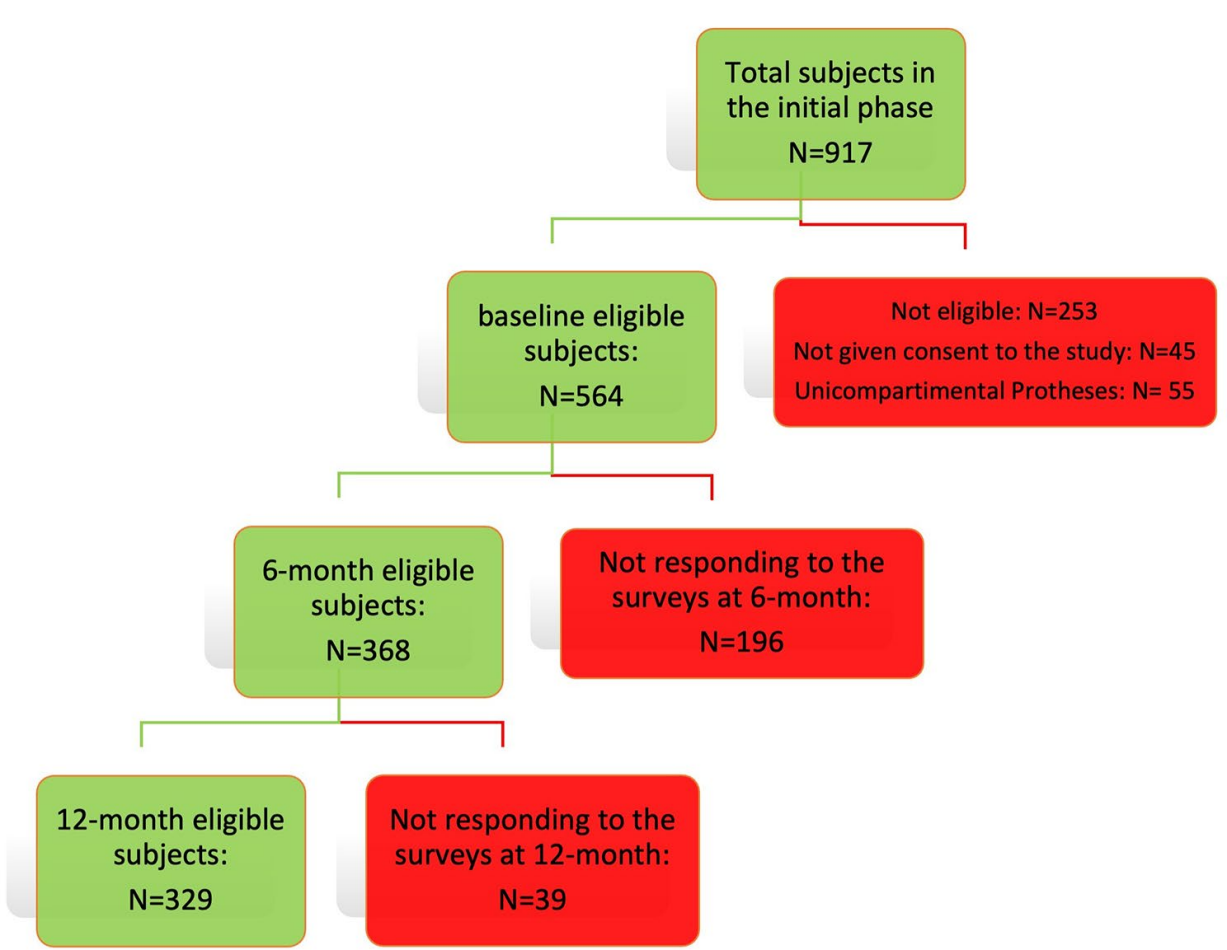
Flow chart of the study population

During the study period, 917 patients underwent KA at the IOR. After excluding ineligible patients (n = 253), patients who refused to participate in the study (n = 45), and patients who had unicompartmental prostheses (n = 55), the study population (Fig. 1) consisted of 564 patients. Complete PROMs data were available at 6 months for 368 patients (65.2%), and at 12 months for 329 patients (58.3%). The comparisons of the characteristics of completers and non-completers of the 6- and 12-month survey are shown in the Supplementary Table S.1. Patients assessed at 12 months had similar baseline characteristics compared with those who did not complete the 12-month survey, except for lower BMI (p = 0.012) and longer length of hospital stay (7.95 ± 3.00 vs. 7.20 ± 2.37; p = 0.007). Moreover, completers had significantly higher mean baseline scores than non-completers on PROM measures: mean EQ-5-3LD 0.47 ± 0.22 vs. 0.42 ± 0.21 (p = 0.016; mean KOOS-PS 50.51 ± 16.43 vs. 46.11 ± 17.65 (p = 0.004).

The mean age was 68.8 years (SD = 9.0, range 32–92) and 67.9% were female. Overall, the mean preoperative PROMs were: 0.45 (SD = 0.22) for the Italian version of the EQ-5D-3 L score, 53.2 (SD = 17.1) for the EQ-VAS, and 48.7 (SD = 17.1) for the KOOS-PS.

The correlations of baseline PROMs ranged from rho = 0.49 (EQ-VAS with EQ-5D-3 L) to rho = 0.53 (EQ-5D-3 L with KOOS-PS), which can be interpreted as medium to large according to Cohen’s conventions [27]. These figures indicate an overlap in content between the two instruments because, in fact, the EQ-5D-3 L score includes the mobility domain.

For the patients included, complete PROMs data were available at 6 months for 368 patients (65.2%), and at 12 months for 329 patients (58.3%).

The mean postoperative PROMs at 6 and 12 months were 0.73 (SD = 0.22) and 0.76 (SD = 0.21) for the EQ-5D-3 L score, 73.1 (SD = 15.4) and 75.3 (SD = 15.5) for the EQ-VAS, and 69.8 (SD = 14.7) and 70.8 (SD = 15.4) for the KOOS-PS.

Table 1 presents the baseline patient characteristics.

**Table 1:** Demographic and clinical characteristics of the study population at baseline (n = 564)

		*n*	%	Mean	SD
**Mean Age**, *y*				68.8	9.0
**Sex**,					
	Female	*383*	63.9		
	Male	*181*	32.1		
**BMI**, *n (%)*					
	normal weight/underweight	99	17.6		
	overweight	*232*	41.1		
	Obese	*233*	41.3		
**Diagnosis**					
	Osteoarthritis	*412*	73.0		
	Other	*152*	27.0		
**ASA score**					
	1	*45*	8		
	2	*382*	68		
	3	*137*	24		
**Length of stay**, days		*7*	6–8		
M-CDS*		*n*	%		
	0–1	*73*	22.1		
	2–4	*211*	63.9		
	5–6	*46*	14.0		
**PROMs baseline score**					
	EQ-5D-3 L			0.45	0.22
	*EQ-VAS*			53.2	17.1
	KOOS-PS			48.7	17.1
**PROMs 6-month score**					
	EQ-5D-3 L			0.73	0.22
	*EQ-VAS*			73.1	15.4
	HOOS-PS			69.8	14.7
**PROMs 12-month score**					
	EQ-5D-3 L			0.76	0.22
	*EQ-VAS*			75.3	15.5
	HOOS-PS			70.8	15.4

Note: * M-CDS = Modified-Chronic Disease Score. Available only for patients residing in RER, Emilia Romagna region (N = 330)

### Model selection and characterization of trajectories

LCGA were used to determine the number of trajectories for two PROM measures of quality of life and functioning.

**Fig. 2:**
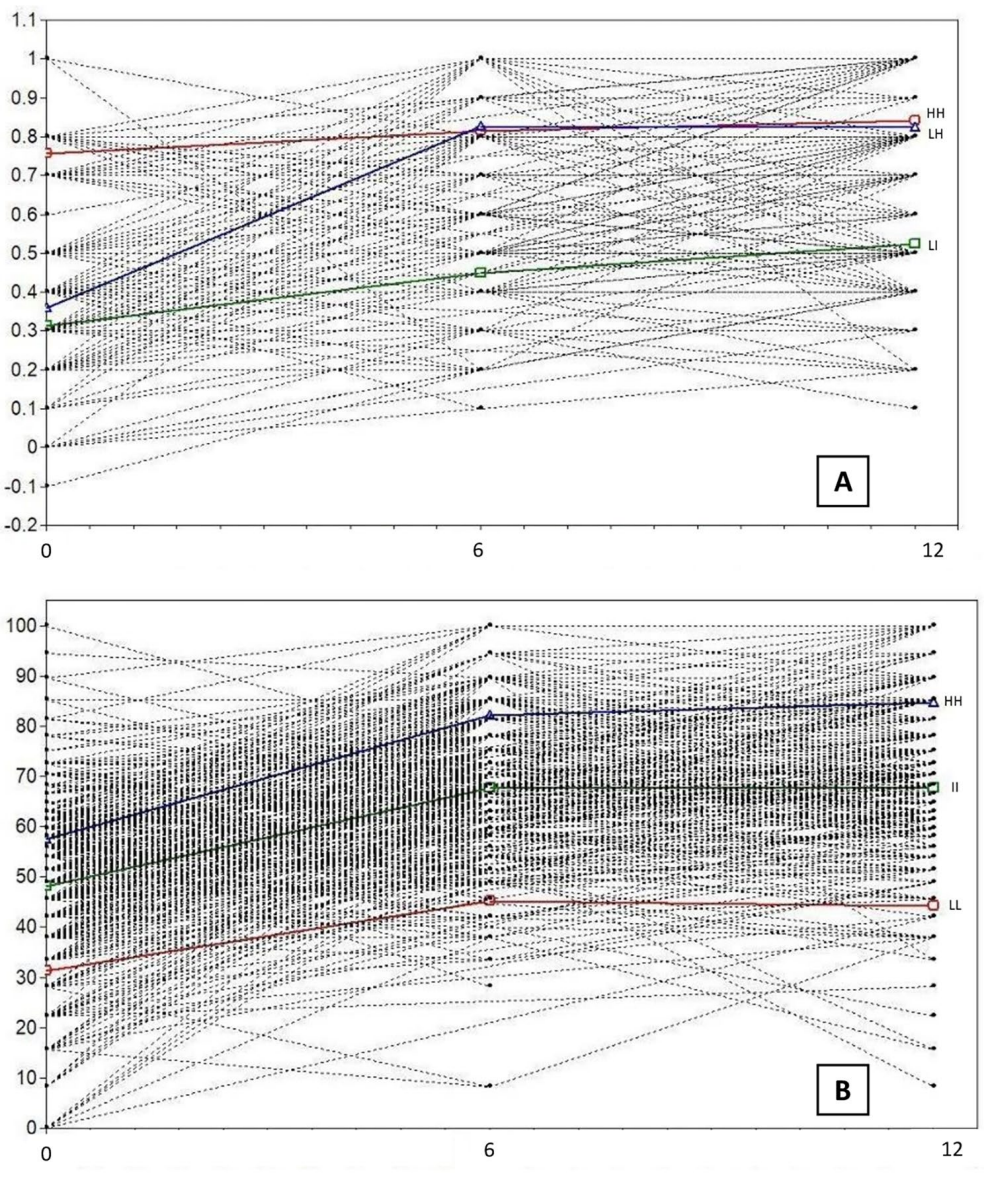
Spaghetti plots of individual trajectoriess for EQ-5D-3 L (A) and KOOS-PS (B) scores, and estimated trajectories using Latent Class Growth Analysis. HH, High-High PROMs trajectory; II, Intermediate-Intermediate PROMs trajectory; LH, Low–High PROMs trajectory; LL, Low-Low PROMs trajectory

The LCGA model with 3 classes was selected as the best performing over the 2-class one (Supplementary Tables S.2. and S.3.). The 3-class model produced three distinct trajectories, each with at least 3% of cases in the smallest class. Complete and detailed information can be found in the supplementary material. Figure 2 depicts a spaghetti plot with the individual trajectories for the three PROMs measures and the trajectories estimated using GMM.

*EQ-5D-3 L trajectories*. The first trajectory included 155 (27.5%) patients with higher pre-surgery EQ-5D-3 L scores, improving at 6 months, and maintaining a stable score at 12 months (high-high trajectory, HH). The second trajectory included 291 (51.6%) patients with low EQ-5D-3 L scores, strongly improving at 6 months, and remaining stable at 12 months (low-high trajectory, LH). The third trajectory included a group of 118 (20.9%) individuals with low scores at baseline, and slightly improving at 6 and 12 months (Low-Intermediate trajectory, LI).

*KOOS-PS trajectories*. The first trajectory included 68 (12.0%) patients with higher pre-surgery KOOS-PS scores, improving at 6 months, and maintaining a stable score at 12 months (high-high trajectory, HH). The second trajectory included a group of 342 (60.6%) individuals with intermediate KOOS-PS scores, improving at 6 months and then stabilizing at 12 months (intermediate-intermediate trajectory, II). The third trajectory included 154 (27.4%) patients with low KOOS-PS scores, improving at 6 months, and stabilizing thereafter (low-low trajectory, LL).

Patients were cross-classified according to the trajectory group for *EQ-5D-3 L* and *KOOS-PS* to determine whether the results were consistent across PROMs indicators (Supplementary Tables S.6. and S.7). 414 patients were placed in the best categories across the two indicators (HH and LH for the EQ-5D-3 L, and HH and II for the KOOS-PS). Only 36 patients were placed in the worst trajectory group for both indicators (LI and LL respectively). An analysis of the subgroup of patients who exhibited inferior outcomes as measured by both the KOOS-PS and EQ-5D-3 L scales, in comparison to the remaining participants in the sample (as presented in Table S.7), revealed that these 36 had higher ASA scores and had a higher chance of having a BMI higher than 30.

### Socio-demographic and clinical characteristics of patients by trajectory groups

*EQ-5D-3 L trajectories (*Table 2*)*. Patients assigned to the LH trajectory based on posterior probabilities estimated by GMM were more likely to be female (OR = 1.943, 95% CI 1.215–3.108, p = 0.006) compared to those in the HH trajectory. Patients in the LI trajectory group were more likely to be female (OR = 2.423, 95% CI 1.339–4.385, p = 0.003) and to have a higher ASA score (OR = 2.027, 95% CI 1.066–3.854, p = 0.031) compared to those in the HH trajectory.

**Table 2:** Results of the multinomial logistic regression for EQ-5D-3 L

Covariates	LH vs. HH			LL vs. HH		
	Odds ratio	95% CI	*p* value	Odds ratio	95% CI	*p* value
**Sex**						
Females	1.943	1.215–3.108	**0.006**	2.423	1.339–4.385	**0.003**
Males	–	–	**–**	–	–	–
**Age**	0.981	0.955–1.008	0.167	0.981	0.950–1.013	0.248
**BMI**						
Obese	0.685	0.343–1.368	0.284	0.779	0.355–1.707	0.532
Overweight	0.624	0.319–1.221	0.169	0.493	0.221-1.100	0.084
Normal weight/underweight	–	–	–	–	–	–
**ASA score**						
3	1.347	0.761–2.385	0.306	2.027	1.066–3.854	**0.031**
<3	–	–	**–**	–	–	–
**Diagnosis**						
Primary Osteoarthritis	1.021	0.605–1.725	0.937	1.113	0.592–2.092	0.741
Other diagnosis	–	–	–	–	–	–

**Statistically significant results are in boldface**

**HH, High-High PROMs trajectory; LL, Low-Low PROMs trajectory; LH, Low–High PROMs trajectory**

*KOOS-PS trajectories (*Table 3*)*. Patients assigned to the LL trajectory based on posterior probabilities estimated by GMM were more likely to be female (OR = 4.215, 95% CI 1.833–9.693, p = 0.001) younger (OR = 0.941, 95% CI 0.896–0.989, p = 0.016), and have a higher ASA score (OR = 4.916, 95% CI 2.074–11.654, p < 0.001), compared to those in the HH trajectory. Conversely, patients in the II trajectory group were more likely to be female (OR = 5.536, 95% CI 2.486–12.330, p < 0.001) compared to those in the HH trajectory.

**Table 3:** Results of the multinomial logistic regression for KOOS-PS

Covariates	LL vs. HH			II vs. HH		
	Odds ratio	95% CI	*p* value	Odds ratio	95% CI	*p* value
**Sex**						
Females	4.215	1.833–9.693	**0.001**	5.536	2.486–12.330	**< 0.001**
Males	–	–	–	–	–	–
**Age**	0.941	0.896–0.989	**0.016**	0.961	0.920–1.003	0.069
**BMI**						
Obese	2.514	0.917–6.887	0.073	1.427	0.553–3.680	0.462
Overweight	0.853	0.273–2.665	0.784	1.498	0.594–3.776	0.392
Normal weight/underweight	–	–	–	–	–	–
**ASA score**						
3	4.916	2.074–11.654	**< 0.001**	1.240	0.546–2.814	0.607
<3	–	–	**–**	–	–	–
**Diagnosis**						
Primary Osteoarthritis	0.669	0.294–1.525	0.339	0.933	0.435–2.004	0.860
Other diagnosis	–	–	–	–	–	–

**Statistically significant results are in boldface**

**HH, High-High PROMs trajectory; II, Intermediate-Intermediate PROMs trajectory; LL, Low-Low PROMs trajectory**

The VIF ranged from 1.01 to 1.25, raising no concerns about multicollinearity.

## Discussion

This study modeled the empirical patterns of quality of life and functioning among patients who underwent TKA, and the characteristics related to each pattern.

Overall, the baseline characteristics and PROMs scores of the study population, both before and after surgery, are in line with previous studies, although data were collected at slightly different time points [28, 29]. In fact, this study has an important strength in the collection of PROMs at 6 months after surgery, which is different from other studies that examined longer-term outcomes (at 12 or 24 months). Therefore, it provides insight into the medium-term effect of the surgical intervention on PROMs.

The largest proportion of patients in this study had low-intermediate PROMs scores at the time of surgery, moderately improved at 6 months, and maintained adequate levels of performance at 12 months post-surgery. Although the analysis shows three main trajectories for the two PROMs instruments, the patterns of these trajectories were slightly different across them. Indeed, they investigate different aspects of the perceived health status [30]. The EQ-5D-3 L measures health-related quality of life and consists of five dimensions: mobility, self-care, usual activities, pain/discomfort, and anxiety/depression. Hence, people reporting higher scores for this scale tend to benefit from more autonomy in activity of daily living, less pain, and better mental health. The KOOS-PS is a questionnaire that measures the level of function in performing usual daily activities (such as rising from bed), and higher-level activities related to the knee joint (such as squatting). Patients with higher KOOS-PS scores tend to benefit from higher functionality and mobility.

The results of the multinomial logistic regression analysis revealed the patient characteristics associated with different trajectories of the two PROMs tools.

The findings concerning the EQ-5D-3 L score indicate two trajectories with low baseline scores, with one of them (low-high) showing a significant improvement 6 months, starting from lower average levels of quality of life at the time of undergoing TKA surgery. This trajectory is the one that describes the pattern of improvement of the majority of patients, confirming the efficacy of this surgical intervention. The results also confirm the current evidence which shows that about 20% of patients undergoing TKA do not show improvements in quality of life [31–34]. Specifically, Bourne and colleagues [31] reported that, among their study sample, satisfaction with pain relief varied from 72 to 86% and with function from 70 to 84% for specific activities of daily living, supporting these results.

Moreover, the findings of this study also showed that females reported lower levels of perceived quality of life before surgery than males. There is evidence suggesting that being female is associated with poorer clinical conditions and perceived quality of life prior to TKA [35], prompting them to seek care. Plenty of literature [36–39] reports that individuals who present late to knee arthroplasty surgery may have reduced gait and biomechanics, as well as a scarce functional recovery after surgery. One study by Lee and colleagues [40] found that patients who underwent knee arthroplasty at a later stage of their disease had a significantly worse preoperative gait pattern compared to those who had the surgery earlier. Additionally, these individuals also had a less favorable postoperative outcome in terms of both function and knee joint biomechanics. Lee’s study suggests that patients who present later in the disease process may have developed compensatory mechanisms, such as limping or favoring one leg, which can negatively impact their gait and overall recovery post-surgery. These findings reinforce the available evidence that early intervention for knee arthritis and other knee conditions may be important to prevent the development of compensatory mechanisms, maintain muscle mass and strength, and ultimately improve the outcome of knee arthroplasty surgeries.

Furthermore, the results suggest that being female is associated with better outcomes after surgery [41]. In fact, women may be more likely to adhere to post-surgical rehabilitation protocols and have better overall health behaviors [35]. Additionally, women may be more likely to have access to support networks and social resources, which can help improve their overall well-being and recovery after surgery [42, 43]. It is also important to note that the relationship between gender, presentation to knee arthroplasty surgery, and outcomes after surgery is complex and may be influenced by a variety of factors.

The results also indicate that patients with an ASA score ≥ 3 were more likely to report lower quality of life at baseline and not to derive significant benefit from surgery at 6 and 12 months (low-intermediate trajectory). The ASA score is indeed a widespread tool used to assess patients’ eligibility for surgery.

The study findings confirm the importance of the ASA score for categorizing patients at different levels of risk also after surgery [44–46].

The ASA score, combined with other indicators, could eventually be used to predict a patient’s expected quality of life improvement after TKA, thus allowing patients and surgeons to make the most appropriate choice, relying on a routinely used tool.

As for KOOS-PS score, findings suggest that knee functionality tends to show gradual and almost uniform improvement in all 3 trajectories. The main difference among these three trajectories is that while the HH and II trajectories, representing 82% of the sample, showed a clinically significant improvement of KOOS-PS (> 10 points [47]) at 12 months, the LL trajectory failed to achieve a clinically significant improvement, denoting the presence of a subgroup of patients who benefit to a minor extent from surgery. Indeed, patients assigned to the low baseline functioning trajectory failed to achieve the minimal important change of ten points in KOOS-PS suggested by Macri et al. [47] and constitute therefore an important target for improvement. Therefore, the main finding of this study is the identification of 3 trajectories of functioning, 2 characterized by moderate improvement after surgery and then stabilization, and 1 by a modest improvement with respect to the low baseline level.

Female patients were more likely to exhibit both the trajectories characterized by lower scores at baseline and at subsequent time points.

Younger patients also showed a slightly worse improvement (LL trajectory). Recent evidence supports these findings stating that being younger is associated with worse outcomes after knee arthroplasty [48]. Specifically, this systematic review by Keeney and colleagues reported worst outcomes in younger patients (under 55 years) [48]. One potential explanation for this association is that younger individuals may be more active and have higher functional demands, which can put more strain on the implanted prosthetic joint and increase the risk of complications [35]. Additionally, younger patients may have a longer lifespan with the implant, increasing the likelihood of wear and tear on the joint over time [49].

Moreover, patients with a higher ASA score are more likely to present the worst trajectory (LL). It is worth noticing that this is the only trajectory not showing a clinically significant improvement 12 months after surgery [47]. Therefore, a high ASA score can be considered a significant determinant of suboptimal recovery after the intervention. The analysis reinforces the body of evidence on the importance of the ASA score for stratifying patients into different levels of functional outcomes after TKA [44–46].

Overall, the analysis confirmed the recent evidence on the variability in long-term pain and function trajectories after total knee replacement [28, 50]. Specifically, most patients tend to have an improvement in pain and function during the first year post-operative; especially in the first 6 months [50].

In summary, this analysis highlighted differential patterns of improvement after TKA. There are also slight differences in the factors influencing PROM trajectories, most likely related to the fact that the two PROMs questionnaires investigate two domains, i.e., quality of life, and joint functioning and mobility. Female gender appears to be associated with a presentation to surgery with worse perceived quality of life and joint function than males, but also more rapid improvement after surgery. Having an ASA score greater than 3 is instead associated with a worse functional recovery after TKA. While a BMI higher than 30 was not found to be significantly associated with the worse single trajectory in the multinomial logistic regression of each PROMs score, the cross-classification between the performance trajectories showed that it was related to the small share of worse performing patients in both the PROMs tools.

### Study limitations

The study cohort was recruited from a large, specialized tertiary care hospital in Italy that is a recognized center of excellence for orthopedic and bone pathologies. As a result, the findings of this study are based on a selected patient sample. Due to the observational nature of this study, these findings are only generalizable to individuals meeting the same inclusion/exclusion criteria. Therefore, additional research conducted in diverse patient populations and healthcare settings is required to validate and expand upon the conclusions.

Additionally, the results may be biased by patient dropout. Specifically, patients who were lost to follow-up differed in certain characteristics (e.g., BMI) from those who were evaluated at 6 and 12 months, which could at least partially undermine the internal validity of the study. However, the dropout rate is comparable to those reported in other comparable studies [24, 51–55]. In addition, it is possible that unmeasured variables (e.g., educational level, socio-economic status, ethnicity) could also be relevant to the missing data process at follow-up [56]. Furthermore, the use of PROMs evaluation at only three time points restricted the ability to detect early improvements or deteriorations or more complex patterns of change. The broad confidence intervals for some of the comparisons were a result of the small number of patients in certain subgroups, which reduced the statistical power to detect significant differences.

## Conclusions

In conclusion, the present study identified three distinct trajectories of PROMs in patients undergoing elective knee arthroplasty. One trajectory, characterizing the best outcomes, was common to both the PROMs instruments used. Being female, younger, and having a higher ASA score were found to be associated with the worst trajectory in the PROMs questionnaire investigating joint functionality and mobility, and, therefore, limited improvement after surgery. However, it is important to note that the evidence for these associations is not yet conclusive and further studies are needed to confirm these findings.

Overall, this study highlights the importance of using multiple PROMs instruments to better understand patterns of patient outcomes after knee arthroplasty and identify factors that may influence these outcomes. Future research should focus on developing strategies to improve PROMs trajectories and optimize outcomes for all patients undergoing elective knee arthroplasty. This study adds to the growing body of literature on the use of PROMs in the assessment of surgical outcomes and may inform the development of interventions to improve the care of patients undergoing knee arthroplasty.

## Electronic supplementary material

Below is the link to the electronic supplementary material.

Supplementary Material 1

## Data Availability

The data used in the study are controlled by Rizzoli Orthopedic Institute and cannot be shared publicly. However, aggregated, and anonymized data are available upon specific request to the corresponding authors. Interested researchers can replicate our study findings by contacting the authors or the Rizzoli Orthopedic Institute.

## Abbreviations

AIC: Akaike Information Criteria
ASA: American Society of Anesthesiologists
BIC: Bayesian Information Criterion
BMI: Body mass index
ECI: Elixhauser comorbidity index
EQ-5D-3L: Euro Quality 5 Dimensions 3L
GMM: Growth Mixture Modeling
HH: High-high trajectory
II: Intermediate-intermediate trajectory
ICD: International Classification of Diseases
ISOC: International Society of Orthopaedic Centers
IOR: IRCCS Rizzoli Orthopedic Institute
KOOS-PS: Knee injury and Osteoarthritis Outcome Score Patient Satisfaction
LCGA: Latent class growth analysis
LH: Low-high trajectory
LI: Low-Intermediate trajectory
LL: Low-low trajectory
MAR: Missing at random
M-CDS: Modified chronic disease score
OECD: Patient-reported outcome measures
RIPO: Registry of Orthopedic Prosthetic Implants
TKA: Total knee arthroplasty
