# Supplementary material for: Identifying patient subgroups with different trends of patient-reported outcomes (PROMs) after elective knee arthroplasty
Source: BMC Musculoskelet Disord. 2023 Jun 3;24:453. doi: 10.1186/s12891-023-06373-2 (PMC10239199; doi:10.1186/s12891-023-06373-2)
Supplement: Supplementary file 1 — Supplementary Material 1 [file 12891_2023_6373_MOESM1_ESM.docx]

**Supplemental material**

**Missing data treatment information**

We employed Full Information Maximum Likelihood (FIML), to estimate missing data of outcome variables. Missing values of covariates were not imputed. Missing data patterns are represented in Figure S1.

**Model selection**

The initial analysis of the frequency distribution of scores for the KOOS-PS revealed a normal distribution, whereas the EQ-5D-3L exhibited a bi-modal distribution, indicating a violation of the assumption of normality for the latter scale. As a result, a latent-class mixed-effect model was employed to estimate continuous non-Gaussian outcomes for the EQ-5D-3L. However, the results obtained were consistent with those obtained under the assumption of normality. Consequently, the manuscript presents results based on the assumption of normality for the two Patient-Reported Outcomes Measures (PROMs). Several different covariance structures, including autoregressive, Brownian motion, and unstructured, were tested, and the results were found to be similar. The results presented in the manuscript assume an unstructured covariance structure.

The posterior probability of class membership for each allocated class was found to range between 0.85 and 0.97 for the EQ-5D-3L, and between 0.79 and 0.85 for the KOOS-PS (as presented in Supplementary Tables S.4. and S.5.). All indicators for the KOOS-PS were consistent with a three-class solution, whereas for the EQ-5D-3L, the model providing the most clinically interpretable description of PROMs patterns was selected. The four-class models were rejected as they did not result in a better fit to the data.

Subsequently, a Gaussian Mixture Model (GMM) was estimated, in which the intercept and slopes were allowed to vary within classes (Supplementary Tables S.4. and S.5.).

**Table S.1. Patients' characteristics. Comparison between completers vs lost to 12-month follow-up.**

|  | **Completers, N = 329** | **Lost to 12-month follow-up, N = 235** | **p-value** |
| --- | --- | --- | --- |
| **Age, mean±SD** | 69.33±8.74 | 68.02±9.30 | 0.15 |
| **Sex** |  |  | 0.12 |
| Males | 114 (35%) | 67 (29%) |  |
| Females | 215 (65%) | 168 (71%) |  |
| **BMI** |  |  | 0.012 |
| Normal weight/underweight | 58 (18%) | 41 (17%) |  |
| Overweight | 151 (46%) | 81 (34%) |  |
| Obese | 120 (36%) | 113 (48%) |  |
| **Diagnosis** |  |  | 0.6 |
| Other | 86 (26%) | 66 (28%) |  |
| Primary arthrosis | 243 (74%) | 169 (72%) |  |
| **ASA score** |  |  | >0.9 |
| *1* | 18 (7.7%) | 27 (8.2%) |  |
| *2* | 158 (67%) | 224 (68%) |  |
| *3* | 59 (25%) | 78 (24%) |  |
| **Length of stay, days** | 7.20±2.37 | 7.95±3.00 | 0.007 |
| **M-CDS*** |  |  | 0.2 |
| 0-1 | 41 (21%) | 32 (24%) |  |
| 2-4 | 132 (67%) | 79 (59%) |  |
| 5-6 | 23 (12%) | 23 (17%) |  |
| *Missing* | 101 | 133 |  |
| **EQ-5D-3L** | 0.47±0.22 | 0.42±0.21 | 0.016 |
| **KOOS-PS** | 50.51±16.43 | 46.11±17.65 | 0.004 |

**Note: *** M-CDS=Modified-Chronic Disease Score. Available only for patients residing in RER, Emilia Romagna region (N = 330)

**Table S.2. Goodness of fit parameters of the LCGA and GMM models for KOOS-PS.**

| Model | Classes | | LL | npar | AIC | BIC | Entropy | A-LRT | p-value |
| --- | --- | --- | --- | --- | --- | --- | --- | --- | --- |
| LGCA | | 1 | -5552.652 | 6 | 11117.304 | 11143.314 | - | - | - |
| LCGA | | 2 | -5455.704 | 10 | 10931.407 | 10974.758 | 0.659 | 186.535 | 0.0016 |
| LCGA | | 3 | -5416.676 | 14 | 10861.352 | 10922.043 | 0.670 | 75.092 | 0.0423 |
| LCGA | | 4 | -5396.045 | 18 | 10828.089 | 10906.120 | 0.662 | 39.697 | 0.0760 |
| LCGA | | 5 | -5376.858 | 22 | 10797.716 | 10893.087 | 0.679 | 36.917 | 0.3452 |

| GMM | 3 | -5334.022 | 26 | 10720.044 | 10832.755 | 0.730 | 38.201 | 0.2398 |
| --- | --- | --- | --- | --- | --- | --- | --- | --- |

Labels: LL = Log Likelihood, npar = Number of Free Parameters, AIC = Akaike’s Information Criterion, BIC = Bayesian Information Criterion, L2 = Likelihood Ratio Chi-Square, A-LRT = Lo-Mendell-Rubin Adjusted Likelihood Ratio Test. LCGA = latent class growth analysis, GMM = growth mixture model.

**Table S.3. Goodness of fit parameters of the LCGA and GMM models for EQ-5D -3L.**

| Model | Classes | LL | npar | AIC | BIC | Entropy | A-LRT | p-value |
| --- | --- | --- | --- | --- | --- | --- | --- | --- |
| LCGA | 1 | 157.746 | 6 | -303.493 | -277.483 | - | - | - |
| LCGA | 2 | 265.966 | 10 | -511.932 | -468.582 | 0.701 | 208.222 | <0.001 |
| LCGA | 3 | 307.153 | 14 | -586.306 | -525.615 | 0.770 | 79.246 | 0.0130 |
| LCGA | 4 | 334.517 | 18 | -633.033 | -555.002 | 0.704 | 52.650 | 0.0519 |
| LCGA | 5 | 358.378 | 22 | -672.756 | -577.385 | 0.807 | 65.226 | 0.0648 |
|  |  |  |  |  |  |  |  |  |
| GMM | 3 | 370.851 | 26 | -689.702 | -576.990 | 0.771 | 159.566 | 0.2395 |

Labels: LL = Log Likelihood, npar = Number of Free Parameters, AIC = Akaike’s Information Criterion, BIC = Bayesian Information Criterion, L2 = Likelihood Ratio Chi-Square, A-LRT = Lo-Mendell-Rubin Adjusted Likelihood Ratio Test., LCGA = latent class growth analysis, GMM = growth mixture model.

**Table S.4. Latent Class Growth analysis class membership probability for EQ-5D-3L by row.**

| **Class** | **1 (n=155)  High-High** | **2 (n=291)  Low-High** | **3 (n=118)  Low-Low** |
| --- | --- | --- | --- |
| **1** | 0.966 | 0.027 | 0.006 |
| **2** | 0.021 | 0.855 | 0.124 |
| **3** | 0.003 | 0.102 | 0.894 |

**Table S.5. Latent Class Growth analysis class membership probability for KOOS-PS by row.**

| **Class** | **1 (n=68)  High-High** | **2 (n=154)  Low-Low** | **3 (n=342) Intermediate-Intermediate** |
| --- | --- | --- | --- |
| **1** | 0.854 | 0.004 | 0.143 |
| **2** | 0.001 | 0.812 | 0.187 |
| **3** | 0.062 | 0.151 | 0.786 |

**Table S.6. Patients cross-classification according to the trajectory group for KOOS-PS and EQ-5D-3L.**

| **​​** | | **EQ-5D-3L** | | |
| --- | --- | --- | --- | --- |
|  |  | ***HH*** | ***LH*** | ***LL*** |
| **KOOS-PS** | ***HH*** | 65 | 82 | 7 |
|  | ***II*** | 83 | 184 | 75 |
|  | ***LL*** | 7 | 25 | 36 |

**Table S.7. Comparison between the 36 patients cross-classified in the worst LCGA classes and the rest of the sample.**

|  | **Other patients, N = 528** | **Worst performing, N = 36** | **p-value** |
| --- | --- | --- | --- |
| **Age, mean±SD** | 68.84±8.96 | 67.97±9.55 | 0.6 |
| **Sex** |  |  | 0.6 |
| Males | 360 (68%) | 23 (64%) |  |
| Females | 168 (32%) | 13 (36%) |  |
| **BMI** |  |  | 0.002 |
| Normal weight/underweight | 95 (18%) | 4 (11%) |  |
| Overweight | 225 (43%) | 7 (19%) |  |
| Obese | 208 (39%) | 25 (69%) |  |
| **Diagnosis** |  |  | 0.6 |
| Other | 141 (27%) | 11 (31%) |  |
| Primary arthrosis | 387 (73%) | 25 (69%) |  |
| **ASA score** |  |  | <0.001 |
| *1* | 43 (8.1%) | 0 (0%) |  |
| *2* | 366 (69%) | 14 (39%) |  |
| *3* | 119 (23%) | 22 (61%) |  |
| **Length of stay, days** | 7.49±2.70 | 7.92±2.26 | 0.085 |
| **M-CDS*** |  |  | 0.005 |
| 0-1 | 68 (22%) | 5 (24%) |  |
| 2-4 | 203 (66%) | 8 (38%) |  |
| 5-6 | 38 (12%) | 8 (38%) |  |
| *Missing* | 219 | 15 |  |
